# Validity and Reliability of a Tool for Determining Appropriateness of Days of Stay: An Observational Study in the Orthopedic Intensive Rehabilitation Facilities in Italy

**DOI:** 10.1371/journal.pone.0050260

**Published:** 2012-11-21

**Authors:** Aida Bianco, Domenico Flotta, Francesca Lotito, Carmelo G. A. Nobile, Claudia Pileggi, Maria Pavia, for The Collaborative Working Group

**Affiliations:** Department of Health Sciences, University of Catanzaro ‘Magna Græcia’, Catanzaro, Italy; Indiana University and Moi University, United States of America

## Abstract

**Objectives:**

To test the validity and reliability of a tool specifically developed for the evaluation of appropriateness in rehabilitation facilities and to assess the prevalence of appropriateness of the days of stay.

**Methods:**

The tool underwent a process of cross-cultural translation, content validity, and test-retest validity. Two hospital-based rehabilitation wards providing intensive rehabilitation care located in the Region of Calabria, Southern Italy, were randomly selected. A review of medical records on a random sample of patients aged 18 or more was performed.

**Results:**

The process of validation resulted in modifying some of the criteria used for the evaluation of appropriateness. Test-retest reliability showed that the agreement and the *k* statistic for the assessment of the appropriateness of days of stay were 93.4% and 0.82, respectively. A total of 371 patient days was reviewed, and 22.9% of the days of stay in the sample were judged to be inappropriate. The most frequently selected appropriateness criterion was the evaluation of patients by rehabilitation professionals for at least 3 hours on the index day (40.8%); moreover, the most frequent primary reason accounting for the inappropriate days of stay was social and/or family environment issues (34.1%).

**Conclusions:**

The findings showed that the tool used is reliable and have adequate validity to measure the extent of appropriateness of days of stay in rehabilitation facilities and that the prevalence of inappropriateness is contained in the investigated settings. Further research is needed to expand appropriateness evaluation to other rehabilitation settings, and to investigate more thoroughly internal and external causes of inappropriate use of rehabilitation services.

## Introduction

In recent years, developed countries have faced a growing demand for rehabilitation services, attributable to the ageing population and the increasing prevalence of disability due to technical advances in acute healthcare.

The changed healthcare needs of the population and the growing costs related to rehabilitation services utilization have stimulated a growing interest in research aimed at the investigation of effectiveness of post-acute care [1]–[3] and appropriateness [4]–[7] of these services.

It should be pointed out, however, that there is substantial heterogeneity in rehabilitation interventions according to diagnoses and types of patients. Clinical factors, complications and comorbidities, and physical and cognitive functioning should influence the selection of the appropriate care path for rehabilitation. For instance, patients undergoing orthopedic surgery may need rehabilitation care that can be different from patients needing pulmonary rehabilitation. Indeed, psychiatric disorders necessitate of specific and distinctive rehabilitation interventions. In Italy, post-acute rehabilitation refers to the care provided after a disabling event has occurred. Depending on the type of care provided, post-acute rehabilitation is set up at three different levels of care: the intensive rehabilitation (three or more hours a day of therapy), the high-specialty intensive rehabilitation (e.g., spinal unit), and the extensive rehabilitation (at least one hour but less than three hours a day of therapy). It can be delivered in a variety of healthcare settings, ranging from dedicated wards in hospitals providing also acute care to long-term care facilities. Each of these facilities can provide one or more levels of rehabilitation, and the aim is to provide rehabilitation care that best suites patient needs in the most appropriate setting.

In the past, well-designed research has investigated appropriateness of services utilization in various settings, such as adults acute hospital care [8], [9], pediatric [10] and elderly care [11], and in emergency units [12], due to the availability of a widely used and validated tool for the assessment of inappropriate acute hospital use, the Appropriateness Evaluation Protocol (AEP) [13], and its Italian version (PRUO).

The evaluation of appropriateness of post-acute rehabilitation still remains challenging and unexplored. Reasons are mainly due to the particular case-mix of patients accessing rehabilitation and to the lack of a common set of terms and definitions for this particular type of care. So far, these factors have hampered the development of a universal tool for the evaluation of appropriateness in rehabilitation. However, a group of French authors has recently developed the first tool aimed at evaluating the appropriateness of hospitalizations in rehabilitation facilities that involves the use of explicit, objective and standardized criteria.

To our knowledge, this tool has never been used in the Italian context, and information on the appropriateness of rehabilitation services utilization is very scarce in our country, although, according to the Ministry of Health, these facilities are facing a dramatic increase in their use and in the related costs for the National Health Service [14], [15].

Within the vast and heterogeneous demands of rehabilitation care, patients who undergo orthopedic interventions provide a unique opportunity to test a tool for the evaluation of appropriateness, since they are a homogeneous population and represent a substantial proportion of subjects seeking intensive rehabilitation care.

Therefore, our study was restricted to orthopedic intensive rehabilitation in order to assess the rate of appropriateness of the days of stay and to test the validity and reliability of this tool in our context.

## Materials and Methods

### Study Population

Two hospital-based rehabilitation wards providing intensive rehabilitation care located in the Region of Calabria, Southern Italy, were randomly selected among all of the orthopedic rehabilitation facilities accredited by the Regional Health System.

A review of medical records on a random sample of patients aged 18 or more who were admitted to the selected rehabilitation facilities during the period March 2010 through July 2011 was carried out. The analysis was performed on random index days, which were preselected on a monthly basis. Patients admitted or discharged within 24 hours from the index day and all the outpatients were excluded from the analysis.

The following data were recorded for each patient: socio-demographic characteristics (age, sex, marital status, educational level, working status, distance from patient's home to the hospital), information related to hospitalization (date and diagnosis of admission, previous admissions in the same rehabilitation facility, length of stay prior to index day, comorbidities in order to calculate the Charlson index [16]), and the evaluation of patient’s autonomy in performing the basic activities of daily living (BADL) at admission. According to the opinion of a physiatrician, patients were considered autonomous in performing BADL if the Functional Independence Measure (FIM) or the Barthel Index (BI) scored >90 or >75, respectively.

### Review Tool

To determine the appropriateness of days of stay, we used the tool that was specifically developed and validated by Guilé et al. [5] for the evaluation of appropriateness of hospital days in rehabilitation facilities. The evaluation tool and related user manual were requested to and obtained from the authors and were translated with the help of a French native-speaking physician. The tool includes a list of 16 items based on the clinical condition of the patient and care management parameters. Ticking at least one criterion is sufficient to consider the day of stay as appropriate, whereas if none of the criteria is fulfilled the days of stay are considered inappropriate. Moreover, it includes override options for the exceptional situation that allows a reviewer to judge hospital days as appropriate even when none of the above criteria were met.

At the time that the present study was designed and conducted the released tool included only the criteria for the evaluation of appropriateness of hospital days in rehabilitation facilities, and it did not include the reasons accounting for inappropriate days of care. Nevertheless, an attempt to draw a roster of potential reasons of inappropriateness and alternatives that allow the description of factors potentially responsible for unnecessary rehabilitation care was made.

### Validity and Reliability

#### Cross-cultural translation

The appropriateness criteria had been subjected to a cross-cultural translation and adaptation process into the Italian language. The methods to translate the items in the original tool from French (the original version) to Italian and to adapt the appropriateness criteria to the Italian context followed published guidelines [17]. The process of cross-cultural adaptation involved several steps: translation from French to Italian; establishment of an expert committee included two experienced researchers on physical medicine and rehabilitation, three methodologists proficient in survey design, questionnaire development and cross-validation method, one language professional and one translator; first meeting of the expert committee to produce the first Italian draft; pilot-testing on a focused group of patients; second meeting of the expert committee to produce a new consensus version; back-translation to French; re-evaluation by the committee members.

Then, a pilot study was conducted in a convenience rehabilitation facility in order to test the final draft of the tool.

### Content Validity

Subsequently, the translated version of the tool was submitted to a group of experts who were senior researchers in public health and in physical medicine and rehabilitation. They reviewed the format and content of the items, as well as the content validity of the tool as a whole. Eventual disagreement between physicians in reviewing medical records was resolved by discussion with the researcher in physical medicine staying in the facility.

### Test-retest Reliability

Two physicians, who were not involved in patient care and acquainted with the user manual, concurrently and independently reviewed 85 medical records with the aim of evaluating the inter-rater reliability.

### Statistical Analysis

The overall agreement and the *k* statistic were used to assess the inter-rater reliability regarding the appropriateness of days of stay. Univariate and multiple logistic regression analyses were performed to explore the association of patients’ characteristics with the inappropriateness of hospital days. Univariate analysis was carried out using the appropriate test (chi-square test or t-test). Explanatory variables included in the multiple logistic regression model were the following: gender (0 = female, 1 = male), age (continuous in years), marital status (0 = married, 1 = other), distance from patient’s home to the rehabilitation facility (continuous in kilometers), length of stay from admission to index day (continuous in days), patient’s autonomy in performing BADL (0 = not autonomous, 1 = autonomous), Charlson comorbidity index (0 = none comorbidity, 1 = Charlson comorbidity index ≥1). Adjusted odds ratio (OR) and 95% confidence intervals (CI) were calculated. The significance level was set at a *P*-value ≤0.05. All the analyses were programmed in Stata software program release 11.0 [18].

The Ethics Committee of the Mater Domini Hospital of Catanzaro (Italy) approved the protocol of the study (Prot. E.C. No. 03/CE/2010). Written consent was requested during the hospitalization, and only the patients who had given written permission for their personal data to be stored in the hospital database and used for research were included in the study.

## Results

The process of establishing content validity resulted in several modifications. The items were reduced from 16 to 11 to adapt their use to the Italian intensive rehabilitation setting. For instance, palliative care and vital sign monitoring were considered to be best suitable in facilities that are classified as high-specialty intensive rehabilitation. The criterion considering the type of rehabilitation professional and the duration of rehabilitation care was modified because it was not considered consistent with the Italian guidelines, which consider days of stay in intensive rehabilitation as appropriate if rehabilitation professionals provide care for at least three hours per day. Thus, the taking over criterion focused on capturing the selected process of care according to the local guidelines. In terms of test-retest reliability of the tool, the overall inter-rater agreement was excellent between the two reviewers, since the agreement and the *k* statistic for the assessment of the appropriateness of days of stay were 93.4% and 0.82, respectively.

Since no significant differences in appropriateness of days of stay between the two rehabilitation facilities studied were found, the results are presented in a combined form. A total of 371 patient days was reviewed, and their main characteristics and distribution of inappropriateness of days of stay according to various explanatory variables are presented in *Table 1*. The mean age was 68 years, and the mean length of stay prior the index day was 11 days. At the time of admission 41% of patients had a Charlson comorbidity index of 1 or more, and almost two-thirds of them were autonomous in performing the BADL. Unexpectedly, it has not been possible to retrieve complete data for some of the patients’ socio-demographic characteristics from medical records; thus, we refrained from reporting those variables that were flawed by a large amount of missing values.

**Table 1:** Descriptive characteristics of study population and distribution of inappropriateness of days of stay according to various explanatory variables.

Characteristic	Total patients surveyed			Inappropriate days of stay		Inappropriate days of stay	Appropriate days of stay
	N	(%)	Mean ± SD	N	(%)	Mean ± SD	Mean ± SD
*Sex (371)*							
Female	250	67.4		51	20.4		
Male	121	32.6		34	28.1		
				χ^2^ = 2.7, 1 df, p = 0.098			
*Age, years (371)*			67.8±13.6			66 (±16.2)	68.4 (±12.6)
						t-test = −1.38, 369 df, p = 0.170	
*Marital status (371)*							
Married	244	65.8		55	22.5		
Other	127	34.2		30	23.6		
				χ^2^ = 0.05, 1 df, p = 0.814			
*Patient’s distance home-rehabilitation facility, km (371)*			70.8±153.4			60.6 (±47.5)	73.9 (±172.7)
						t-test = −0.7, 369 df, p = 0.481	
*Day of week of the index day (371)*							
Weekday	270	72.8		60	22.2		
Weekend	101	27.2		25	24.8		
				χ^2^ = 0.27, 1 df, p = 0.606			
*Charlson comorbidity index(371)*							
0	220	59.3		39	17.7		
≥1	151	40.7		46	30.5		
				χ^2^ = 8.22, 1 df, p = 0.004			
*Length of stay prior the index day, days (371)*			11±6.4			11.4 (±7.7)	10.9 (±6)
						t-test = 0.55, 369 df, p = 0.581	
*Patient autonomy (360)*							
No	131	36.4		28	21.4		
Yes	229	63.6		51	22.3		
				χ^2^ = 0.04, 1 df, p = 0.843			

Overall, 22.9% of the days of stay in the sample were judged to be inappropriate. Inappropriate days of stay appears to be evenly distributed according to most of the explanatory variables investigated, except for sex with 28.1% of inappropriateness in males compared to 20.4% in females, age, since younger patients were more likely to have inappropriate days of stay compared to older ones and Charlson Comorbidity Index, since 30.5% of patients with Charlson Comorbidity Index ≥1 had inappropriate days of stay compared to 17.7% of those with Charlson Comorbidity Index = 0; however only in this case the difference was statistically significant at the univariate analysis (χ^2^ = 8.22, 1 df, p = 0.004) (*Table 1*).

*Table 2* illustrates the distribution of frequency of the items used for the evaluation of appropriateness. The top three criteria were the evaluation of patients by rehabilitation professionals for at least 3 hours on the index day (40.8%), specific nurse care (21.3%), recent intercurrent pathology, evolving or not stabilized, that appeared in the course of hospitalization (17.5%). Moreover, override criteria were used to justify the presence of patient in the facility in 2% of days of stay.

**Table 2:** Frequency distribution of criteria for appropriateness of days of stay.

	Item	N*	%*
1	Complex wound dressings	2	0.6
2	Paramedical surveillance at least three times per 24 h of a given parameter on medical prescription	1	0.3
3	Surveillance of medication under direct medical supervision	42	14.5
4	Diagnostic exploration ongoing	9	3.2
5	Specific nursing care	61	21.3
6	Provisional feeding tube or adaptation in progress	1	0.4
7	Invasive medical act on the day	2	0.7
8	Care by a rehabilitation professional for at least 3 hours on index day	118	40.8
9	Patient in pain	9	3.2
10	Ongoing review of recent or non-stabilized loss of autonomy	21	7.3
11	Recent intercurrent pathology, evolving or not stabilized, that appeared in the course of hospitalization	50	17.5
	Override	6	1.9

* Multiple items selection was possible.

The results of multiple logistic regression analysis (*Table 3*) with inappropriate days of stay as the dependent variable showed that inappropriateness, as expected, was significantly higher for those patients presenting a Charlson comorbidity index of 1 or more (OR = 2.8, 95% CI = 1.6–4.88; p<0.0001), for younger (OR = 0.98, 95% CI = 0.96–1, p = 0.024) and male patients (OR = 0.57, 95%CI = 0.32–1, p = 0.049).

**Table 3:** Multivariate analysis for inappropriateness of days of stay.

Variable	OR	95% CI	p
*Outcome: inappropriate days of stay Log likelihood: −178.81; χ^2^ = 21.25 (8 df); p = 0.006, No. of observations = 360*			
Charlson comorbidity index	2.8	1.6–4.88	<0.0001
Age, years	0.98	0.96–1	0.024
Gender	0.57	0.32–1	0.049
Length of stay from admission to index day	1	0.99–1.07	0.113
Patient’s autonomy	0.87	0.5–1.54	0.640
Distance from patient’s home to rehabilitation facility, Km	1	0.99–1	0.683
Marital status	0.91	0.52–1.59	0.746
Day of week of index day	0.98	0.55–1.76	0.945

Among all the potential causes of inappropriateness, the study results showed that the primary reasons accounting for the inappropriate days of stay were social and/or family environment issues (34.1%), health conditions of patient that prevents the provision of rehabilitation care (31.8%), over cautious physician’s attitude (17.6%), and rehabilitation care that could be delivered at a lower level of care (16.5%).

## Discussion

The present study represents the first attempt to assess the appropriateness of days of stay in rehabilitation facilities in Italy by using a tool developed and validated for this specific purpose. The tool is not merely a modified version of the AEP adapted to rehabilitation facilities, and it is intended to be used in the same way as the AEP. It consists of explicit, objective and validated criteria that were validated using a formalized expert consensus method [5].

A version of the tool that was suitable for the Italian intensive rehabilitation was adapted and tested. The tool seems to be feasible to use, reliable, and to have adequate validity. Cross-cultural translation allowed to adapt the local version of the tool to international standards. Measurement of content validity, defined as the degree to which the content of a measurement tool is an adequate reflection of the construct to be measured, was assessed qualitatively and was necessary because of two reasons. First, the appropriateness criteria that had been drawn from the Italian guidelines on rehabilitation activities were included into the final draft of the tool. Second, some of the items from the original tool were not considered to be suitable for the Italian intensive rehabilitation. Content validity was judged to be good by our expert group, and the tool seems to require no further revision. Moreover, the tool showed to be highly reproducible since the agreement between the reviewers was of 93.4% with a *k*-statistic of 0.82. According to the classification of Landis and Koch, the latter value denotes a high level of reliability. This result is close to that obtained by the authors who developed the tool [5], [6].

Comparison across countries should be made cautiously, given the dissimilarities in the provision of rehabilitation care, the different case-mix of patients, and the modification that were made to the tool. For instance, as previously described in Italy rehabilitation activities are organized in three level of care, depending on care needs of patients, namely intensive rehabilitation, high specialty intensive rehabilitation, and extensive rehabilitation [14]. The organization of rehabilitation services in Italy is similar to that existing in USA where rehabilitation is classified as acute rehabilitation, i.e. the therapy provided in inpatient setting for at least three hours daily with nursing support over the 24 hours, and as sub-acute rehabilitation, i.e. a less intensive therapy for those patients who cannot tolerate or benefit from three hours of therapy daily [4]. In France, rehabilitation therapy can be provided in medium-stay units that are represented by rehabilitation centers and sub-acute care units, and the duration of the therapy varies according to specific objectives of functional recovery [5]. The results of the present study showed a prevalence of inappropriateness of the days of stay of 22.9%, and demonstrate that our results are in line with those obtained from other studies that evaluated the appropriateness in rehabilitation facilities. Nevertheless, the authors of the tool used in the present study reported a percentage of non-appropriate days of stay ranging from 42.4% to 44.6% if only the explicit the criteria were considered, but these figures lowered to about 27% if overrides criteria were also taken into account [5]. Moreover, in their second work that has been recently published and listing the criteria for the analysis of causes of inappropriateness, the same authors reported as inappropriate nearly 25% of days of stay [6]. In a study aimed at validating a modified version of the Italian AEP (PRUO) for the evaluation of the appropriateness of admissions and days of stay in rehabilitation facilities, the percentage of inappropriate days of stay was of 26% [19]. Furthermore, in a study conducted in Australian rehabilitation facilities using a utilization review method, Poulos et al. reported 52% of hospital days as inappropriate [7]. Nevertheless, the percentage of inappropriate days of stay is lower than those obtained from our past experiences in evaluating the appropriateness in acute-care hospitals by using the Italian version of the AEP (PRUO), which showed a prevalence of inappropriate days of stay ranging from 39.5% in a population of elderly patients to 75.5% in a population of patients accessing the hospital through the Emergency Department [9], [11], [12].

The results of multivariate analysis showed that some patient characteristics were significantly related to inappropriate days of care. Indeed, younger age was a predictor of inappropriateness of health service use and a similar findings has been reported in previous studies [12], [20]. Moreover, the patient with higher level of comorbidity have an increased risk of inappropriateness day of stay, and a possible explanation is that social factors could account for more inappropriate days of care in patients with chronic conditions that can be managed with effective outpatient care reducing the need of hospitalization after acute condition was resolved.

Reasons accounting for inappropriate days of care were not available when the present study was designed. However, following on our past experience on appropriateness in acute hospitals, a list of potential causes of inappropriateness was arranged. In the meantime, the authors of the original tool have published their list of reasons accounting for inappropriateness [6]. A comparison showed that both lists were almost overlapping. In our study, the reasons accounting for the inappropriate days of stay were social and/or family environment issues, health conditions of patient that prevent the provision of rehabilitation care, overly cautious physician’s attitude in the management of a patient, and rehabilitation care that could be delivered at a lower level of care. These figures are very similar to those reported by Paillé-Ricolleau et al. [6] who reported that a procedure or a service of social nature, as well as external organizational factors, most often accounted for inappropriate days of stay. In our experience, these factors were also related to the overly cautious attitude because of high comorbidity of patients and other related social factors.

The study results should be read in the light of some limitations. First, retrospective data collection may have distorted the actual rate of inappropriateness, since it is influenced by the quality of medical records. Nevertheless, retrospective data collection is a common and accepted method for the evaluation of appropriateness, since many studies based on the AEP were performed by analyzing medical records of discharged patients. Second, data were collected in one Italian region that might not represent all intensive rehabilitation in Italy, and concern about generalizability and comparability of the findings may arise. However, we are confident that the findings of the study may be representative of the area examined and may be referred to the Southern part of our country. Third, the process of adaptation enabled us to trim the tool for evaluating the appropriateness in intensive rehabilitation only. Although this is a limit of the present study, the tool could serve as a canvas to be adapted in other settings by introducing slight and adequate modifications.

In conclusion, the study findings showed that the tool used is reliable and have adequate validity to measure the extent of appropriateness of days of stay in rehabilitation facilities and that the prevalence of inappropriateness is contained in the investigated settings. Further research is needed to expand appropriateness evaluation to other rehabilitation settings, and to investigate more thoroughly internal and external causes of inappropriate use of rehabilitation services.
